# Investigation of Interface Thermal Resistance between Polymer and Mold Insert in Micro-Injection Molding by Non-Equilibrium Molecular Dynamics

**DOI:** 10.3390/polym12102409

**Published:** 2020-10-19

**Authors:** Can Weng, Jiangwei Li, Jun Lai, Jiangwen Liu, Hao Wang

**Affiliations:** 1 College of Mechanical and Electrical Engineering, Central South University, Changsha 410083, China; canweng@csu.edu.cn (C.W.); lijiangwei@csu.edu.cn (J.L.); 15827170706@163.com (J.L.); 2 College of Mechanical and Electrical Engineering, Guangdong University of Technology, Guangzhou 510000, China; 3 Department of Mechanical Engineering, Faculty of Engineering, National University of Singapore, Singapore 117575, Singapore; mpewhao@nus.edu.sg

**Keywords:** micro-injection molding, interface thermal resistance, non-equilibrium molecular dynamics, phonon density of state

## Abstract

Micro-injection molding has attracted a wide range of research interests to fabricate polymer products with nanostructures for its advantages of cheap and fast production. The heat transfer between the polymer and the mold insert is important to the performance of products. In this study, the interface thermal resistance (ITR) between the polypropylene (PP) layer and the nickel (Ni) mold insert layer in micro-injection molding was studied by using the method of non-equilibrium molecular dynamics (NEMD) simulation. The relationships among the ITR, the temperature, the packing pressure, the interface morphology, and the interface interaction were investigated. The simulation results showed that the ITR decreased obviously with the increase of the temperature, the packing pressure and the interface interaction. Both rectangle and triangle interface morphologies could enhance the heat transfer compared with the smooth interface. Moreover, the ITR of triangle interface was higher than that of rectangle interface. Based on the analysis of phonon density of states (DOS) for PP-Ni system, it was found that the mismatch between the phonon DOS of the PP atoms and Ni atoms was the main cause of the interface resistance. The frequency distribution of phonon DOS also affected the interface resistance.

## 1. Introduction

With the rapid developments of micro-electro-mechanical systems (MEMS), parts containing nanostructures can be used in self-cleaning surfaces [1], lab-on-chip devices [2], biomedical detection [3], optical technology [4] and other fields. In recent years, micro-injection molding has aroused extensive research interests to fabricate polymer products with nanostructures due to its advantages of low cost and short cycle [5,6].

During micro-injection molding of nanostructured parts, the interfacial heat transfer between the filled polymer and the mold insert will affect the quality of nanostructures [7,8,9]. It is believed that the interfacial thermal resistance is an important parameter to study the interfacial heat transfer characteristics in micro-injection molding for nanostructures. The “interface” here refers to the interface between the polymer melt and the mold core. And the interface thermal resistance (ITR) is not equal to the thermal contact resistance (TCR). The ITR refers to the thermal resistance of two surfaces in full contact at the atomic scale, while the TCR refers to the thermal resistance caused by the gap between two contact surfaces. The TCR between the polymer and the mold insert was studied by numerical simulation and experimental methods [10,11]. For example, Hong et al. conducted numerical simulation to study the relationship between the filling behavior of polymer melt and TCR in injection molding [11].

The computational fluid dynamics simulation based on continuum mechanics would be failed to accurately explain and predict the ITR between the polymer and the mold insert at the nanoscale. Therefore, the molecular dynamics (MD) simulation method provides the possibility to study the interfacial heat transfer in nanostructures. Pina-Estany et al. studied the heat transfer coefficient (HTC), between the polyethylene and the nickel mold insert in different nanocavity areas. The results showed that increasing the surface-to-volume could improve the thermal transport properties [12]. The study of ITR in injection molding by MD method is rarely reported in the literature. However, the non-equilibrium molecular dynamics method to study the heat transfer characteristics in polymer nanocomposites or microelectronics would provide us inspiration and guidance [13,14,15,16,17]. Ju et al. simulated the ITR of bi-layer nanofilms with different interfacial temperatures, layer thicknesses and mass ratios using MD simulation methods, but in this case, their model built with virtual atoms has limited practical significance [15]. Barisik et al. used MD simulation to analyze temperature dependence of thermal resistance at the water/silicon interface. It was found that interface resistance increased with the increase of temperature for high wetting surface, and the opposite behavior was true for low wetting surfaces [16]. Vo et al. established the MD model to study Kapitza resistance under different solid-liquid interaction intensities and found that the interface thermal resistance decreased with the increase of the solid-liquid interaction intensity [17]. Focusing on the improvement of interfacial thermal transport, Luo et al. investigated the effects of graphene size, interfacial bonding strength, and polymer density on interfacial thermal transport [18]. Zhang et al. studied the ITR between polyethylene (PE) and silicon substrate and found that the increase of temperature and contact strength could significantly improve the interfacial thermal conductivity [19]. Similarly, Hu et al. found that the increase of bonding strength could improve the interfacial thermal conductivity of the silicon-polyethylene interface [20].

The purpose of this paper was to study the heat transfer between the polymer and the mold insert during micro-injection molding. The non-equilibrium molecular dynamics (NEMD) simulation was carried out to investigate the effects of the temperature, the packing pressure, the interface morphology, and the interfacial interaction on the ITR between the polypropylene (PP) layer and nickel (Ni) layer. Furthermore, the phonon density of state (DOS) was introduced to understand the mechanism of interfacial heat transfer.

## 2. Materials and Methods

**Figure 1:**
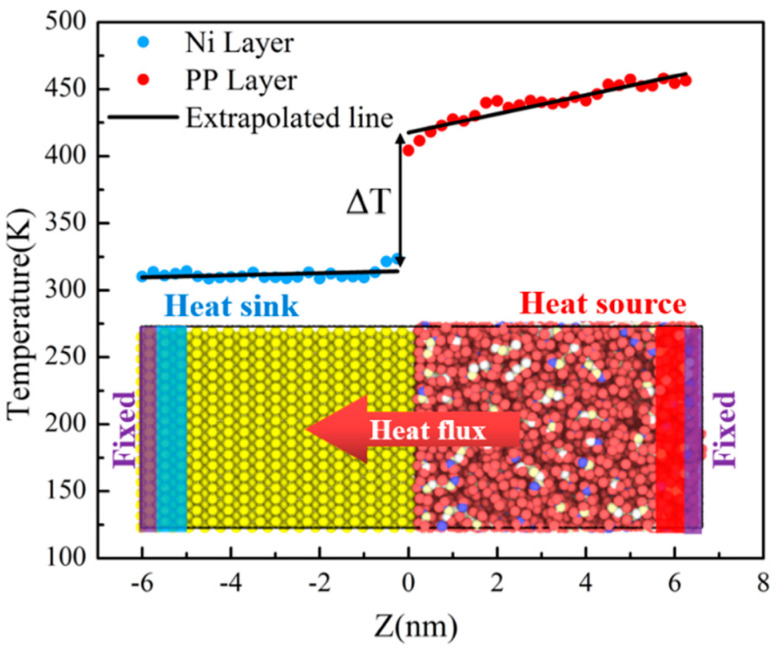
Non-equilibrium molecular dynamics (NEMD) simulation model for the polypropylene-nickel (PP-Ni) system and the temperature profile in a steady state.

The polymer simulated in this study was polypropylene (PP), which has been widely used in the field of automotive lightweight due to its good mechanical properties [21]. The initial density of PP was set to be 0.9 g/cm^3^ at 298 K and 1 atm. There were 150 chains in the box, and the polymerization degree of each chain was 10. In order to eliminate the stress of PP system and keep it in the energy minimization state, it was necessary to conduct energy minimization and cyclic annealing treatment for PP system. The annealing temperature of PP was 528K. Ni was selected as mold insert material, which was constructed by nickel unit cell as FCC structure with (1 0 0) plane. The length and width of the Ni layer were consistent with the dimensions of the PP layer. The interfacial model of Ni-PP system was shown in Figure 1.

The NEMD simulation of all-atom model used the Consistent Valence Force Field (CVFF) to describe the potential of intermolecular interactions [5,22]. The nonbonded interactions between PP molecules and mold insert atoms were described by Lennard-Jones (L-J) potential. The molecular interaction parameters between PP and Ni could be calculated by Lorentz-Berthelot mixing rules (see Equations (1) and (2)). The L-J parameters of different atoms in this study were shown in Table 1. The cut-off distance was 1.25 nm.

(1) $$\varepsilon_{ij}=\sqrt{\varepsilon_{i}\varepsilon_{j}}$$

(2) $$\sigma_{ij}=\left(\sigma_{i}+\sigma_{j}\right)/2$$

where ε and σ are the energy and distance constants respectively. The subscripts *i* and *j* refer to different atom types.

The NEMD simulation method is a powerful technique to generate the temperature gradient and heat flux in structures [23,24,25,26]. As shown in Figure 1, the length of the whole system was about 12.5 nm. There were two fixed layers at both ends of the system, with a thickness of 0.3 nm, to stabilize the free ends and prevent external energy exchange. Then, the heat source and heat sink layers with a thickness of 0.5 nm were set beside the fixed layer by the Nose-Hoover thermostat, respectively. Before the simulation of heat conduction, the whole system reached the thermal equilibrium state at the specified temperature under the NVT ensemble. Next, the NVT ensemble was removed. The heat source and heat sink were applied and extracted energy separately under the NVE ensemble to establish the temperature gradient across the junction. For the whole simulation procedure, the integral time step Δt was 1.0 fs. The total number of simulation steps was 2,000,000, about 2.0 ns. The first 1.5 ns was used for system balance, and the last 0.5 ns was used for analysis. The periodic boundary conditions were applied in the x and y directions. The heat flux was 0.001 Kcal/mol (equal to 6.95 × 10^−24^ J). Finally, the system was equally divided into 50 blocks. When the system reached its steady state, an obvious temperature drop ΔT appeared on the interface. The results showed that there existed an ITR between the PP layer and the Ni layer. The above simulations were completed by LAMMPS [27], an open source molecular dynamics package in a computer cluster with AMD Opteron 6128 processor running in parallel with 40 cores.

The heat flux density J along the *z*-axis can be calculated by Equation (3) [28]:

(3) $$J=\frac{E}{At}$$

where E is the energy imposed on the system, A is the cross-sectional area of the system and t refers to the total simulation time.

The ITR is defined by Equation (4) [17,18]:

(4) $$R=\frac{\Delta T}{J}$$

where the value of ΔT can be obtained from the temperature drop at the interface between the PP layer and the Ni layer.

Thermal energy is the energy of atomic vibrations in nature. From the acoustic mismatch model (AMM) or diffusive mismatch model (DMM), the function of phonons is closely related to the heat conduction [29]. In the vibrations of a three-dimensional lattice, it can be characterized by the phonon DOS in the frequency domain.

The simulation results were analyzed from the point of phonon DOS, which was calculated by taking the fast Fourier transform (FFT) of the velocity autocorrelation functions (VAF) of the atoms (see Equation (5)) [30,31].

(5) $$D\left(\omega \right)=\int_{0}^{\tau }\Gamma \left(t\right)\cos\left(\omega t\right)dt$$

where ω denotes frequency, D(ω) is the phonon DOS at frequency ω, and Γ(t), which is given in Equation (6), is the VAF.

(6) Γ(t)=〈v(t)v(0)〉

**Figure 2:**
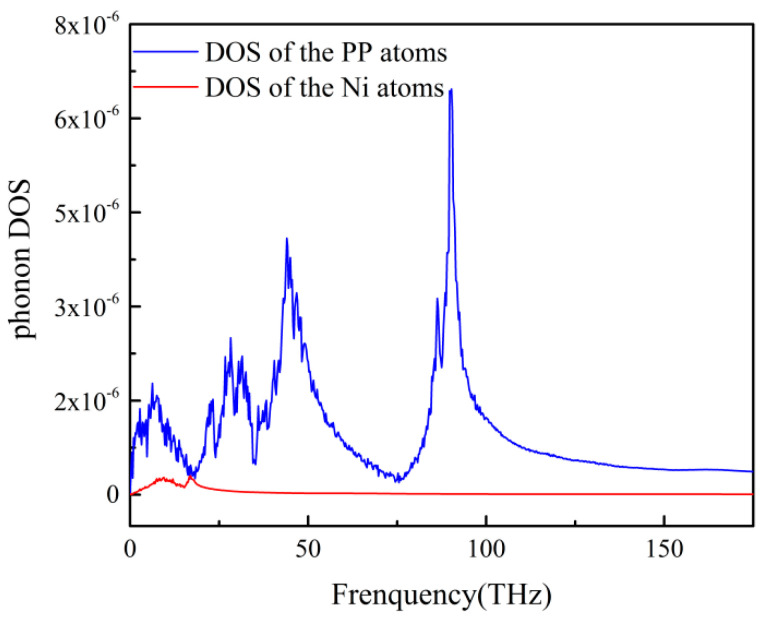
Phonon density of states (DOS) of PP atoms and Ni atoms at 353 K.

A typical DOS of the PP-Ni system was demonstrated in Figure 2. It could be seen that the frequency range of the phonon DOS of PP atoms was about 0–100 THz. The frequency range of phonon DOS of Ni atoms was about 0–20 THz. There was a considerable mismatch between the two groups, indicating that there was a remarkable phonon scattering at the PP-Ni interface. This behavior could clearly explain a high ITR of the PP-Ni system.

## 3. Results and Discussion

### 3.1. ITR at Different Temperatures

**Figure 3:**
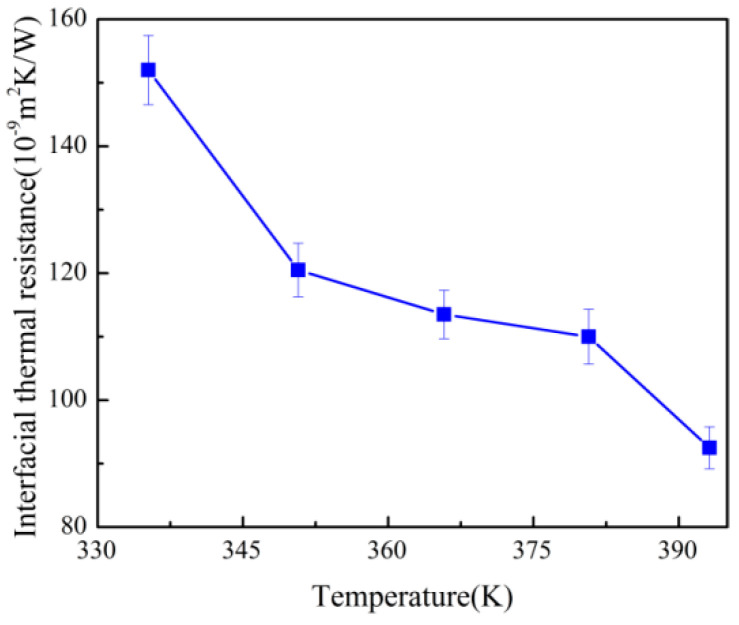
Variations of the interface thermal resistance (ITR) with different temperatures.

Temperature is an important factor affecting the ITR during the injection molding. It can affect not only the fluidity of the polymer melt, but also the motion of phonons. In order to find the relationship between ITR and temperature, at the pressure of 0 MPa, NEMD simulations were carried out at different temperatures of 335 K, 350 K, 365 K, 380 K, and 395 K, respectively. The ITR at different temperatures were illustrated in Figure 3. It could be seen that the ITR between the Ni layer and the PP layer decreased gradually with the increase of the temperature. The ITR was about $1.52\times 10^{-11}\;m^{2}{\mathrm{KW}}^{-1}$ with the IT at 335 K, while it dropped about 39% with the IT at 393.17 K. This change of ITR with the temperature was basically the same as that in Ref [32], but the values of the ITR were about three orders of magnitude smaller than the experimentally measured values reported by Zhu [32] ($3.496\times 10^{-4}\;m^{2}{\mathrm{KW}}^{-1}$). The reason was that the work in the above reference was done in the geometry on the macro-scale, not on the nanoscale as in this work. And it also may be affected by partial contact or bulk disorder in the near-surface region during the experiment [29].

**Figure 4:**
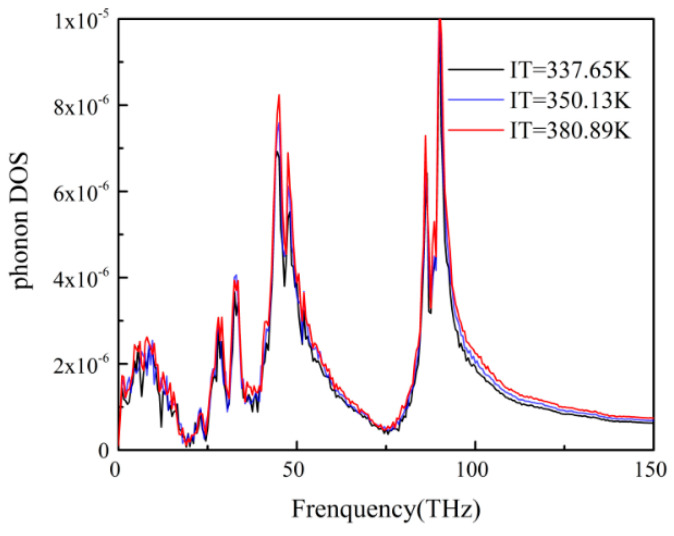
The phonon DOS of PP atoms at different temperatures.

The phonon DOS of the PP layer at three different temperatures was demonstrated in Figure 4. With the increase of temperature, the rate of atomic motion increases. The higher the temperature, the larger the kinetic energy of the PP atom, so the phonon DOS has higher peak intensity. That could explain why the ITR decreased as the temperature increased.

### 3.2. ITR with Different Packing Pressures

**Figure 5:**
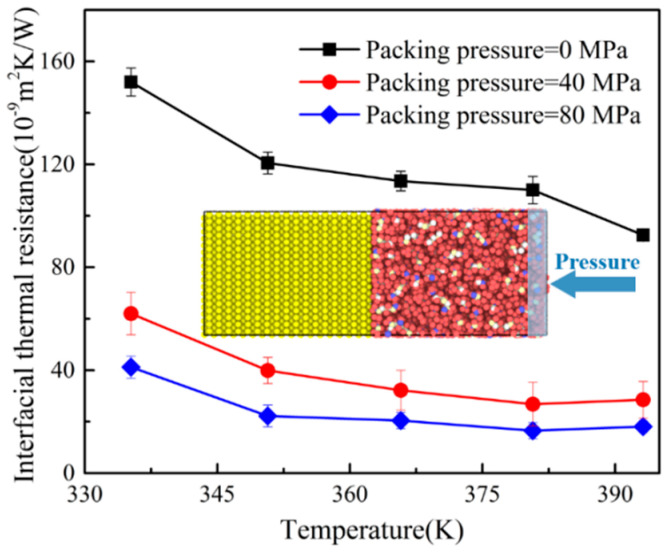
Variations of ITR at the different packing pressures. The inset depicts the PP-Ni system of the applying pressure.

During the injection molding process, the packing pressure would affect the molecular density of polymer. Increasing the packing pressure could make the molecular chains inside the polymer more closely aligned, which may have an impact on the ITR. In this section, the pressure dependency of the ITR between Ni and PP layers was studied. The outermost layer atoms with a thickness of 0.3 nm was applied forces along the −z direction. The force values for each atom were set to $6.9\times 10^{-14}\;N$ and $13.8\times 10^{-14}\;N$, respectively. The applied force was converted to the pressure values of 40.8 MPa and 80.16 MPa, which were common values of packing pressure in micro-injection molding. The packing time was set to 20 ps. The NEMD simulation of PP-Ni system was carried out under different packing pressures. This was compared with the case where the molecules in 3.1 were not under pressure. The ITR at different packing pressures were also calculated. The results were shown in Figure 5 and compared with those in Section 3.1. It was found that the ITR between the PP layer and the Ni layer decreased with the increase of packing pressure. With the temperature of 395 K, the value of ITR was only $1.8\times 10^{-10}\;m^{2}{\mathrm{KW}}^{-1}$ when the packing pressure was 80 MPa, while it decreased by 80.4% under no packing pressure. Therefore, increasing the packing pressure could effectively improve the thermal conductivity between the polymer and the Ni mold insert in the micro-injection molding process.

**Figure 6:**
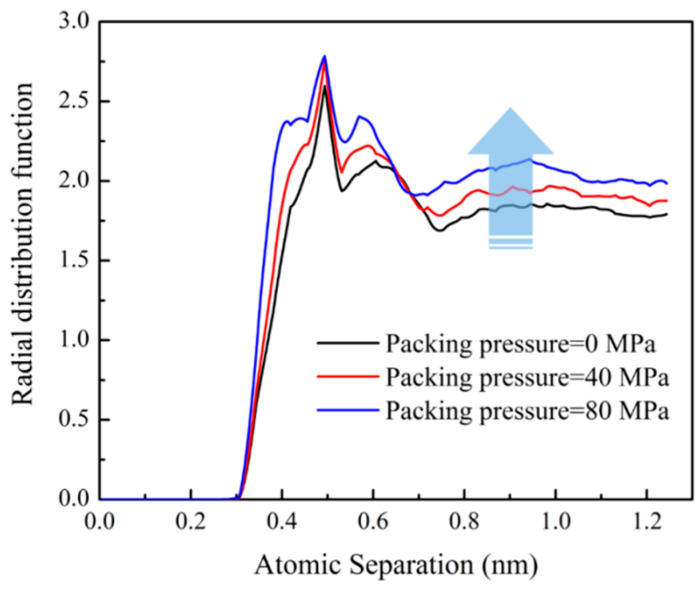
The radial distribution function of carbon atoms in the PP layer with respect to nickel atoms.

**Figure 7:**
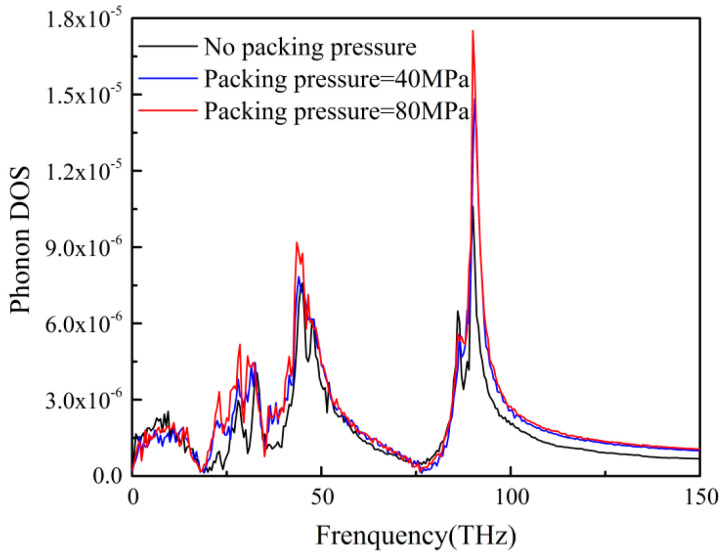
The phonon DOS of PP atoms at different packing pressures.

By changing the packing pressure, the PP system was more compact, and the interatomic distance was reduced. The above effects could be observed by the radial distribution function (RDF) of carbon atoms in PP molecular and nickel atoms. As shown in Figure 6, the RDF of PP atoms increased with the increase of packing pressure, which was consistent with the results reported in some literatures [16,33]. When PP system was compressed, the interatomic distance was shortened. This resulted in more PP atoms interacting with Ni atoms at the same distance. Therefore, there was a stronger effective interaction at the interface between the PP layer and the Ni layer. Similarly, the change of ITR with the packing pressure could also be observed in phonon DOS. The phonon DOS of PP layer under different packing pressures were shown in Figure 7. The phonon DOS with the packing pressure was obviously higher than that with no packing pressure. In the same way, with the increase of packing pressure, the value of DOS increases significantly, which might enhance the heat transfer across the interface.

### 3.3. ITR Under Different Interface Morphologies

**Figure 8:**
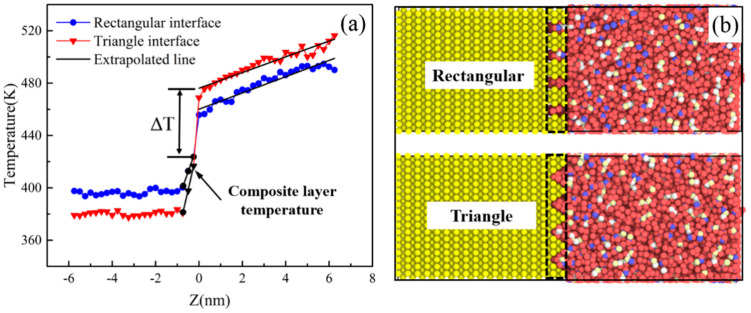
(**a**) The temperature profiles of different interface morphologies; (**b**) rectangular and triangular interface topographies.

**Figure 9:**
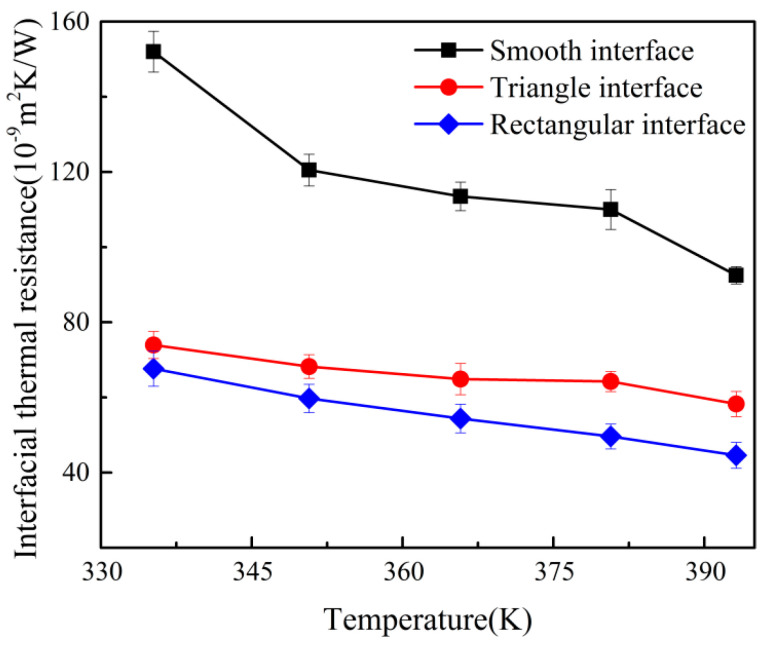
Variations of ITR with different interface morphologies.

**Figure 10:**
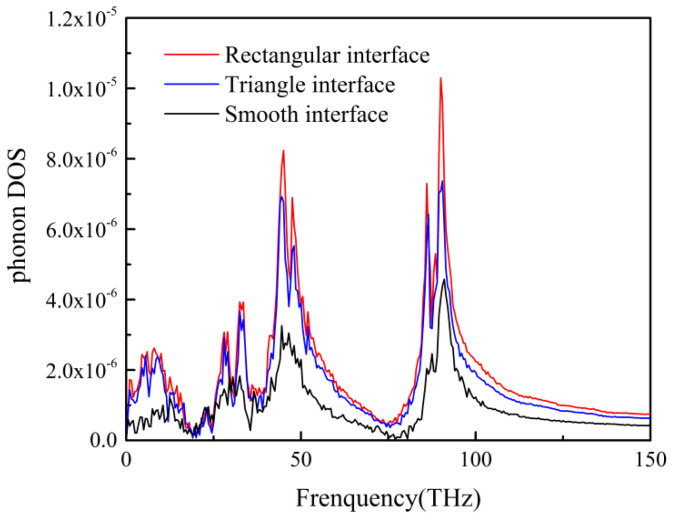
The phonon DOS of PP atoms under different interface morphologies.

The micro-injection molding, the interface between the polymer and the mold insert was usually not smooth. However, the interface morphology of the mold insert may affect the ITR. In this study, two types of interface morphologies were proposed, i.e., the rectangular and the triangular interface morphologies, as shown in Figure 8b. The temperature profiles of different interface morphologies were illustrated in Figure 8a. There were the small temperature gradients inside the composite layer of PP and Ni atoms for both rectangle and triangle interface morphologies. Compared to the smooth interface, the composite layer could reduce the temperature drop of the interface, and the temperature drop was used to calculate the ITR. Also, the temperature drop of rectangle interface was lower than that of triangle interface. A larger temperature drop at the interface represented a larger thermal resistance. The results showed that the thermal resistance of triangular interface was larger than that of rectangular interface. The ITR of different interfacial temperatures for the rectangular and the triangle interfaces was shown in Figure 9. It was interesting to find out that both the rectangular and the triangle interfaces could reduce the ITR, compared with the smooth interface. Meanwhile, the ITR of the triangle interface was higher than that of the rectangular interface. The phonon DOS of PP layer with different interface morphologies were shown in Figure 10. Obviously, the phonon frequencies of the rectangle and the triangle interfaces were higher than that of the smooth interface. This will contribute to the microinjection molding process of surface microstructural products such as grating structure or super hydrophobic structure.

### 3.4. ITR with Different Interfacial Interactions

From the microscopic point of view, the interaction strength of the interface would directly affect the movement behavior of molecules at the interface, and then affect the heat transfer characteristics of the two groups of atoms at the interface [18]. In order to change the contact strength of Ni-PP system in which the interactions were van der Waals forces, the energy constants describing the interfacial van der Waals forces were changed by 2, 3, 4, 5 times of the original value ($\varepsilon_{0}$), respectively.

**Figure 11:**
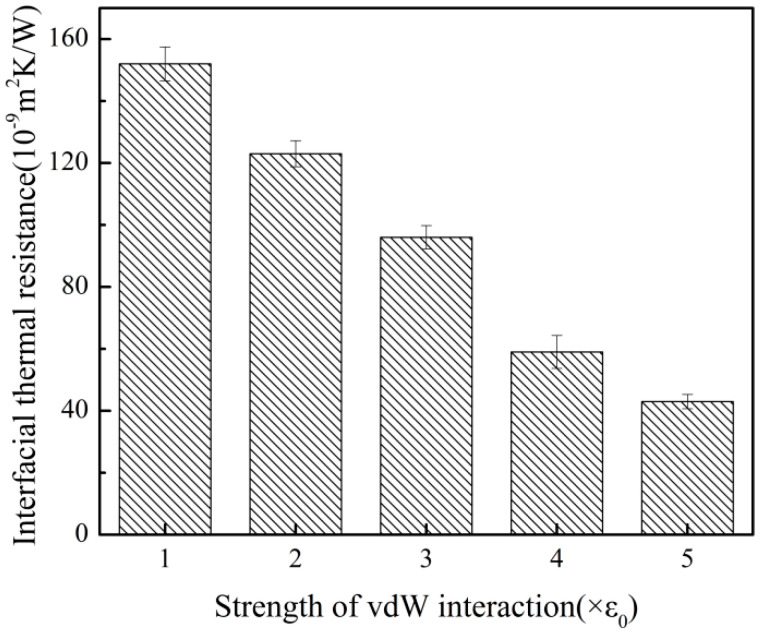
The ITR with different interface interaction strengths.

**Figure 12:**
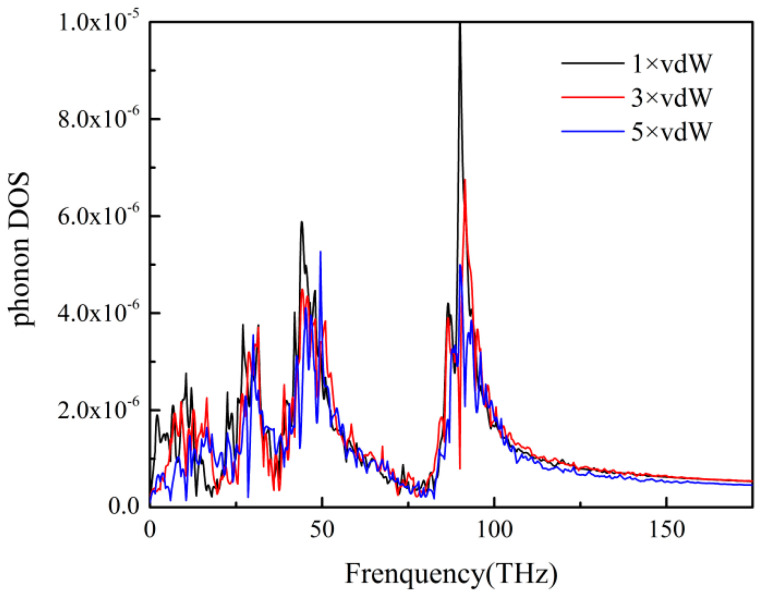
The phonon DOS of PP atoms with different interface interactions.

As shown in Figure 11, the ITR between the PP layer and the Ni layer was weak when the interfacial interaction was strong. The results showed that the heat transfer across PP-Ni interface could be enhanced by increasing the surface energy of mold insert materials in micro-injection molding. Figure 12 depicted the phonon DOS of PP atoms under three interfacial interactions. At the low frequency of 0–20 THz, the matching degree of DOS of nickel atoms and DOS of PP atoms was higher, which resulted in the decrease of ITR for PP-Ni system.

## 4. Conclusions

In this paper, the NEMD simulations for micro-injection molding were carried out to investigate the ITR between the PP layer and the Ni layer. The result showed that the ITR reduced about 24.38% when the temperature increased from 335 K to 393 K. The results of ITR at different packing pressures indicated that the increase of packing pressure could enhance the heat transfer between the PP layer and the Ni layer. In addition, the interfaces of rectangle and the triangle could reduce the ITR compared with the smooth interface. At last, interfacial interaction strength also affected the ITR. The stronger the interactions, the smaller the interface thermal resistance.

The mechanisms of ITR were discussed from the point of phonon DOS of the PP-Ni system. There was a considerable mismatch between the phonon DOS of the two groups, which resulted in the phonon scattering across the interface. This behavior could clearly explain the considerable ITR of the system. For different temperatures, packing pressures, interface morphologies and interface interactions. The peak value of the phonon DOS can better reflect the size of the interface thermal resistance: the larger the peak value, the smaller the interface thermal resistance value will be.

## Figures and Tables

**Table 1:** Lennard–Jones (L-J) potential parameters for different atom types.

Atom Type	Energy Constant *ε* [eV]	Distance Constant *σ* [Å]
H	0.038	2.450
C	0.039	3.875
Ni	11.983	2.282

## Nomenclature

ε	The energy constant
σ	The distance constant
$\sigma_{i,j}$	Distance between the atom *i* and *j*
J	Flux density
E	The energy imposed on the system
A	The cross-sectional area
ΔT	Temperature difference
R	The interface thermal resistance
